# Climate data dynamics: A high-volume real world structured weather dataset

**DOI:** 10.1016/j.dib.2024.111156

**Published:** 2024-11-20

**Authors:** Md Zubair, Md. Nafiz Ishtiaque Mahee, Khondaker Masfiq Reza, Md. Shahidul Salim, Nasim Ahmed

**Affiliations:** a Department of Computer Science and Engineering, Chittagong University of Engineering and Technology, Chittagong, Bangladesh; b Department of Computer Science and Engineering, BRAC University, Dhaka, Bangladesh; c Department of Computer Science and Engineering, Khulna University of Engineering and Technology, Khulna, Bangladesh; d Department of Computer Science and Engineering, Uttara University, Dhaka, Bangladesh

**Keywords:** Meteorology, Time-series, Rainfall, Temperature, Humidity, Sunshine, Wet-bulb globe temperature, Climate Change

## Abstract

The dataset at hand is a unique resource, officially procured from the Bangladesh Meteorological Department, the sole government institution that diligently monitors weather through 35 strategically placed weather stations across the nation. This dataset is a treasure trove of actual data spanning several decades, from the inception of each weather station to the present. It has been meticulously restructured and processed into four (Rainfall, Temperature, Humidity, and Sunshine) key weather parameters, presented in a highly organized and accessible format. This format not only facilitates its use in the machine-learning training process but also opens up avenues for its application in climate research, weather forecasting, and a myriad of other statistical and machine-learning applications.

Specifications Table

**Table:** 

Subject	*Data Science*
Specific subject area	*Data-driven Intelligent Analysis and Modeling in Precision Agriculture, Environmental Science, Machine Learning, etc.*
Data format	Raw data (CSV format), Filtered (data is preprocessed and filtered to enhance its usability for training and testing models)
Type of data	Table
Data collection	Daily real-world weather data with the four (Rainfall (mm), Temperature (°C), Humidity (%), and Sunshine (h)) features of all (35) the weather stations of Bangladesh have been incorporated in the dataset. The data was collected from the Bangladesh Meteorological Department. With extensive preprocessing, we have structured the dataset in a machine-trainable format.
Data source location	Institution: Meteorological Department of Bangladesh Location: Bangladesh Google Map Area URL: https://maps.app.goo.gl/Zd5Z7paN2bpf6KXR9
Data accessibility	Repository name: Mendeley data repository. Data identification number: 10.17632/tbrhznpwg9.1 Direct URL to data: https://data.mendeley.com/datasets/tbrhznpwg9/1
Related research article	M. Zubair, S. Ahmed, A. Dey, A. Das, and M. M. Hasan, “An Intelligent Model to Suggest Top Productive Crops Based on User Location in Context of Bangladesh”, 3rd International Conference on Smart Systems: Innovations in Computing, India, Springer, pp 289–300. 10.1007/978-981-16-2877-1_26 [1]

## 1 Value of the Data

• This dataset has an enormous scope of application. It can be used in weather and climate research, developing data-driven weather models, weather prediction, data science, and other fields where climate data is required. Secondly, we have collected the data from the highest and most reliable authorities. Finally, the dataset is highly structured and ready to use, which is deemed for high-quality research.
• This datasetʼs primary focus groups are academics and researchers in various fields. However, data analysts and machine learning engineers can use it directly in model design. Secondary user groups include weather journalists, crop field supervisors, and anyone interested in climate dynamics.
• Other researchers, such as those in sociological, medical, or energy research, can also use the dataset to infer and correlate unorthodox patterns. For example, a medical researcher can use the data to find correlations between dengue spread and the amount of rainfall.
• The reliability and vastness of the data are undisputed. The Bangladesh Meteorological Department [2] has been collecting data from 35 weather stations across the country, many of which have been collecting data since 1967.

## 2 Background

The world has been facing various adverse effects of climate change, which has turned into a crisis. Asian countries, mainly those located in South and Southeast Asia, are the biggest victims of this crisis. Persistent inclement weather has made livelihoods difficult. Unprecedented weather conditions have perplexed policymakers, researchers, and other stakeholders, making it difficult to find solutions. Floods, riverbank erosion, drought, and cyclones are some examples of calamities the countries in these regions are suffering. In recent years, the persistent heatwave has been a new phenomenon. A large number of people in Bangladesh alone have been direct or indirect victims of the scorching heat since April 2024 [3].

Additionally, the detrimental impacts of high temperatures on crop production pose a significant threat to global food security. Moreover, experts speculate that the heatwave will not go away in the near future but will persist in the coming years [4]. Under these circumstances, high-quality multidisciplinary research is required to study these matters. We prepare this ready-to-use dataset to support multidisciplinary data-driven research in this field.

## 3 Data Description

Collecting weather data from all the weather stations of a country (35 stations in Bangladesh) is quite challenging. The Bangladesh Meteorological Department helped us by providing the raw data. We further preprocessed the dataset. The dataset contains daily weather data with four features (Rainfall, Temperature, Humidity, and Sunshine) from the establishment of the weather station till the year 2023. The dataset has 543,839 instances. The features are composed of time series and numerical data types. The summary of the dataset has been compiled in Table 1.

**Table 1:** Summary (Starting and Ending year of data collection of each station along with sample size and data type) of the weather dataset.

Station	Starting Year	Ending Year	Sample Size	Data Type
Bogra, Chittagong, Coxsbazar, Dhaka, Sylhet	1961	2023	543,839	Time Series, Numerical
Barisal	1967			
Jessore	1969			
Chandpur, Sitakunda, Teknaf	1977			
Mymensingh, Rajshahi, Rangpur	1979			
Bhola, Comilla	1981			
Khulna, Satkhira	1984			
Faridpur, Feni, Hatiya, Ishurdi, Kutubdia, Madaripur, Mcourt, Patuakhali	1985			
Srimangal	1986			
Rangamati, Sandwip, Tangail	1987			
Khepupara	1988			
Dinajpur	1989			
Chuadanga	1999			
Sudpur	2000			
Mongla	2001			
Ambaganctg	2008			

**Fig. 1:**
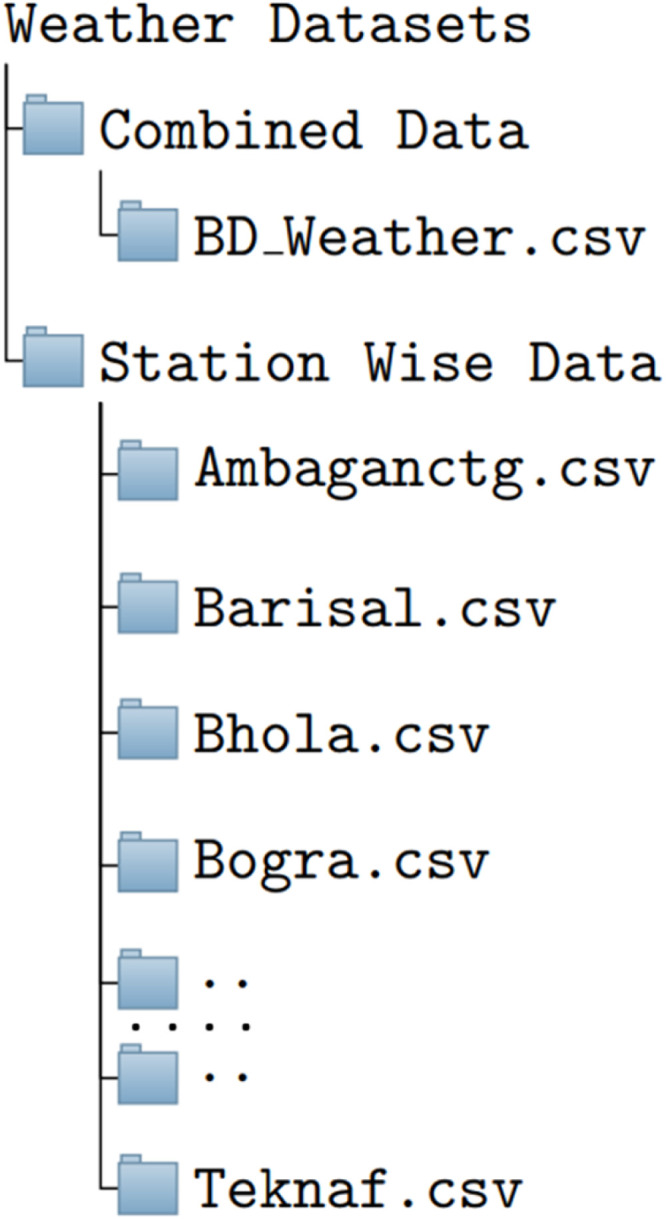
Folder structure of our weather dataset.

We have combined all the four features into a single CSV data file. In the data repository, we have created a folder named **Weather Datasets**. Under the folder, there are two subfolders (*Combined Data and Station wise Data*) [5]. The combined Data subfolder contains the BD_Weather.csv file and the Station Wise Data subfolder has 35 CSV files for each weather station. Fig. 1 represents the folder structure of our dataset.

## 4 Experimental Design, Materials and Methods

We are living in a data-driven world, and it is the fuel of the modern era. Data is used for intelligent system building and decision-making. Weather data is important for climatologists and weather forecast-based system building. Though we have collected the dataset from the meteorological department of Bangladesh, the data was too unstructured to be used. So, we carefully pre-processed the dataset, analyzed it, made a machine-trainable format, and tested our dataset by training a predicted model. We have discussed the steps in the following subsections.

### 4.1 Data preprocessing

**Fig. 2:**
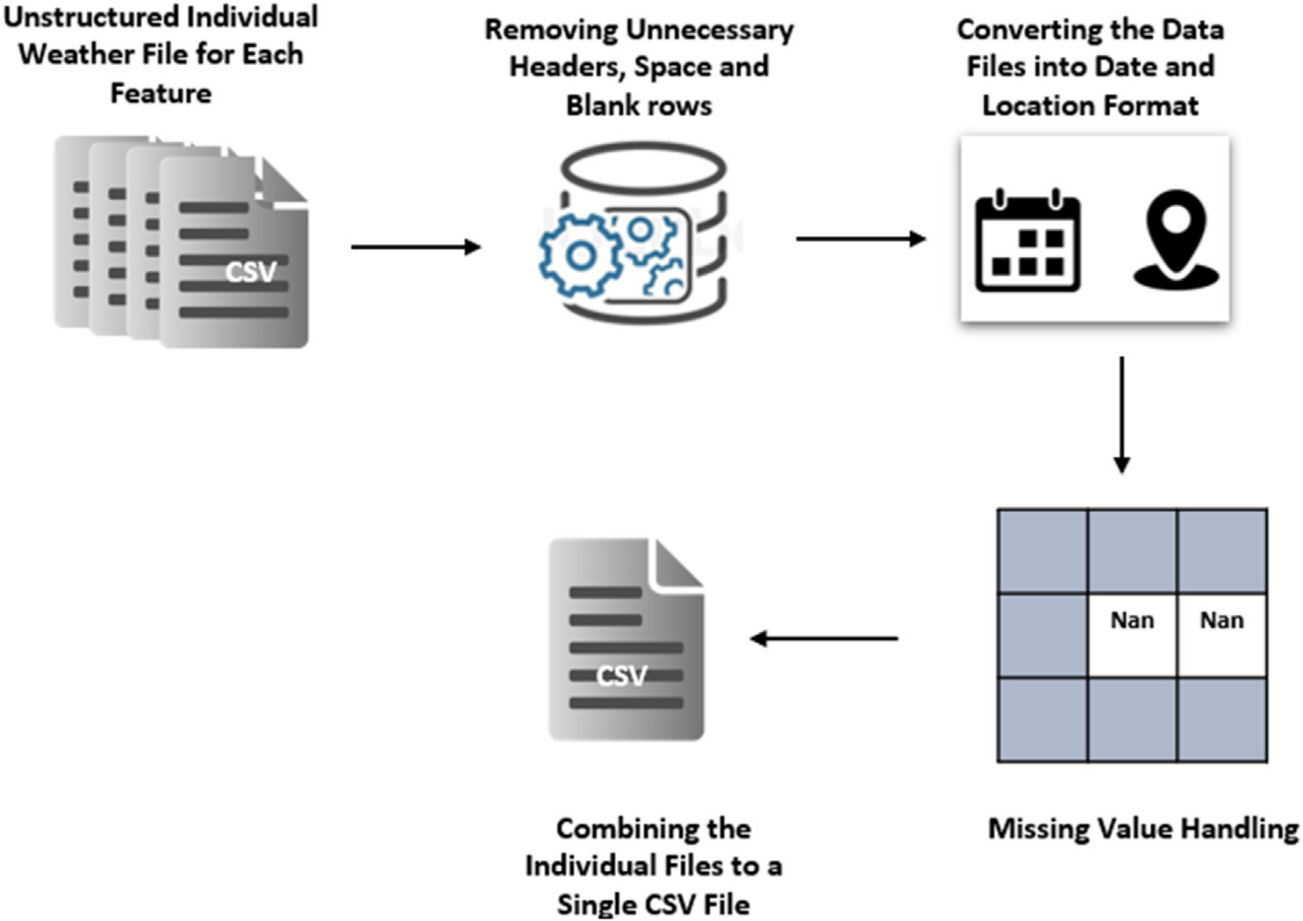
Methodology for preprocessing the weather dataset.

The meteorological department of Bangladesh provided the dataset with four individual CSV files for four features. These files were unstructured containing unnecessary texts, special characters, garbage values, blank rows and columns, and missing values. Our main challenge was to preprocess these datasets. Their preprocessing is graphically represented in Fig. 2.

Data preprocessing consists of five stages: data collection, data cleaning, standardization, handling missing values, and data integration.

**Fig. 3:**
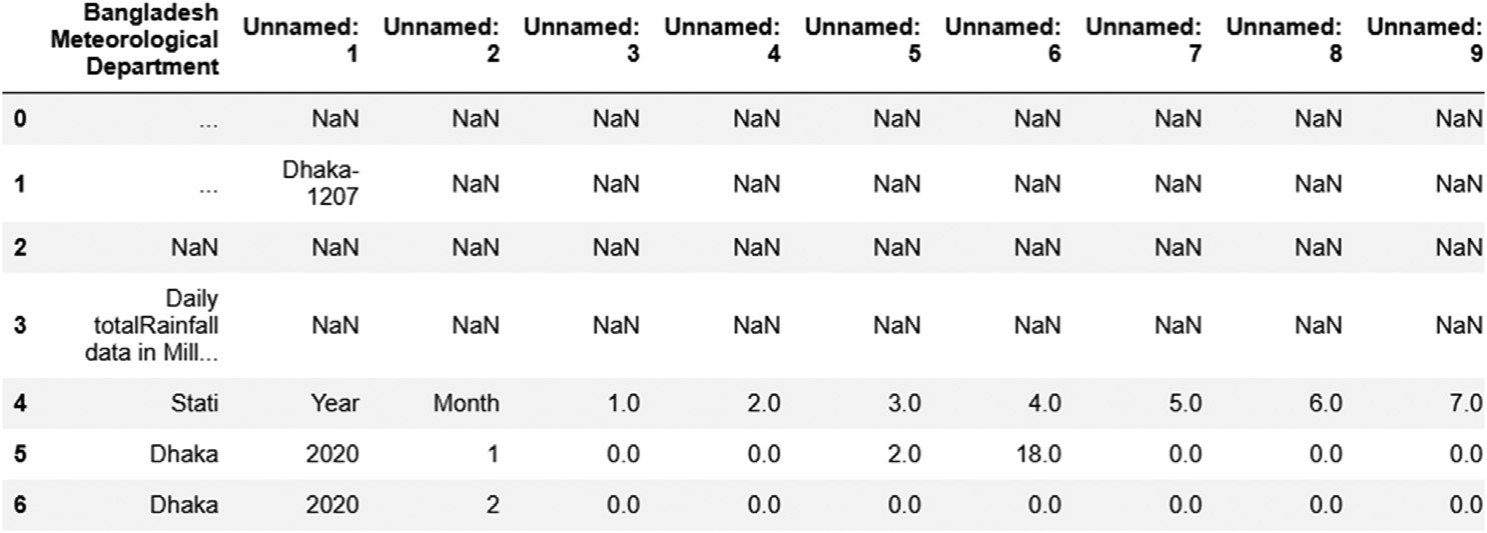
Sample RAW rainfall data of Dhaka weather station.

**Fig. 4:**
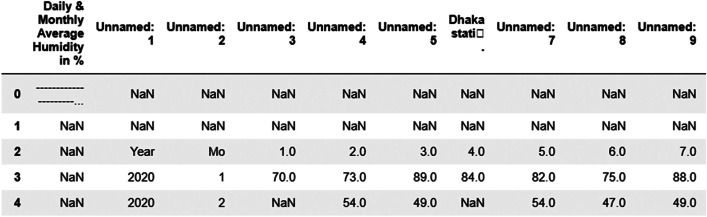
Sample RAW humidity data of Dhaka weather station.

**Fig. 5:**
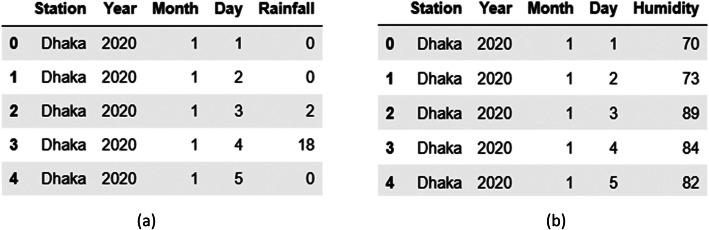
Sample RAW data of Dhaka weather station is converted into a standard format. (a) Rainfall and (b) Humidity.
• *Handling Missing Values:* Missing values caused by device failures are managed using forward and backward filling, mean imputation, linear interpolation, and manual techniques.
• *Data Integration:* Finally, we merged all four data files (rainfall, temperature, humidity, and sunshine) into a single CSV file. Final view of few sample data is given in Fig. 6.

• *Data Collection*: We collected unstructured data on rainfall, temperature, humidity, and sunshine from the Meteorological Department in CSV format on a daily basis for 35 weather stations across Bangladesh.
• *Data Cleaning:* We removed unnecessary literals, such as unnecessary text, headers, spaces, blank rows, and columns, using Python libraries like Pandas and NumPy.
• *Standardization:* Different data files were provided in different patterns. In this step, we converted them into a consistent format. To illustrate, we have shown collected sample data for the Dhaka weather station in Fig. 3 for rainfall and Fig. 4 for humidity. We converted the sample data of Fig. 3, Fig. 4 into a structured format as represented in Fig. 5.

**Fig. 6:**
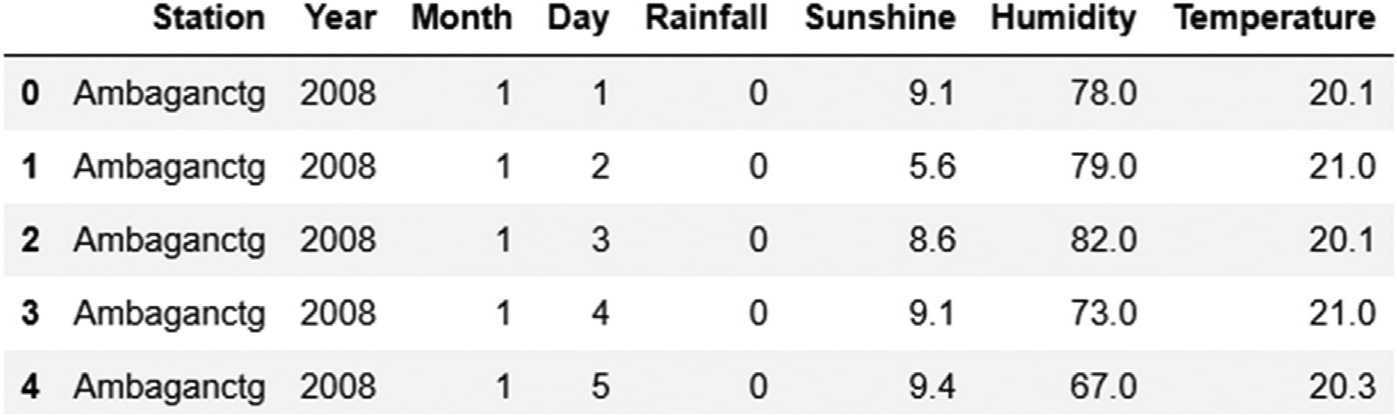
Sample data after preprocessing and concatenating the individual file.

In Algorithm 1, we have provided our data preprocessing procedure for better intuition.

**Algorithm 1:** Data Preprocessing.

1. **Input:** Unstructured RAW weather dataset. 2. **Output:** Structured dataset 3. **Procedure** DATA PREPROCESSING (*Input*) a. Load CSV files for daily rainfall, temperature, humidity, and sunshine data for all stations. b. For each file: - Remove unnecessary elements (text, headers, spaces, blank rows/columns). - Standardize column names and formats. c. For each file: - If format varies, convert to a consistent structure. d. For each column: - Use forward/backward fill for isolated missing values. - Use mean imputation for random missing values. - Use linear interpolation or manual filling as needed. e. Concatenate all cleaned data files into a single dataset and save as a CSV. **End procedure**

Now, our structured and pre-processed weather dataset can be used for weather forecasting and other climate analysis.

### 4.2 Data analysis

**Fig. 7:**
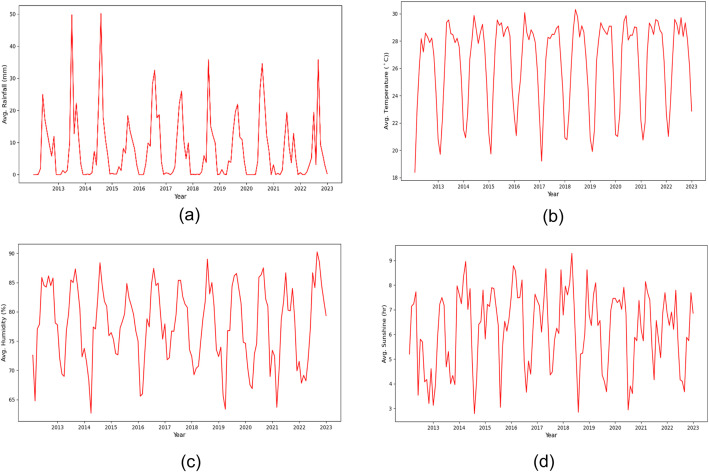
Weather trend of (a) Avg. Rainfall (mm), (b) Avg. Temperature (°C), Avg. Humidity (%) and Avg. Sunshine (hrs) of Dhaka weather station.

**Fig. 8:**
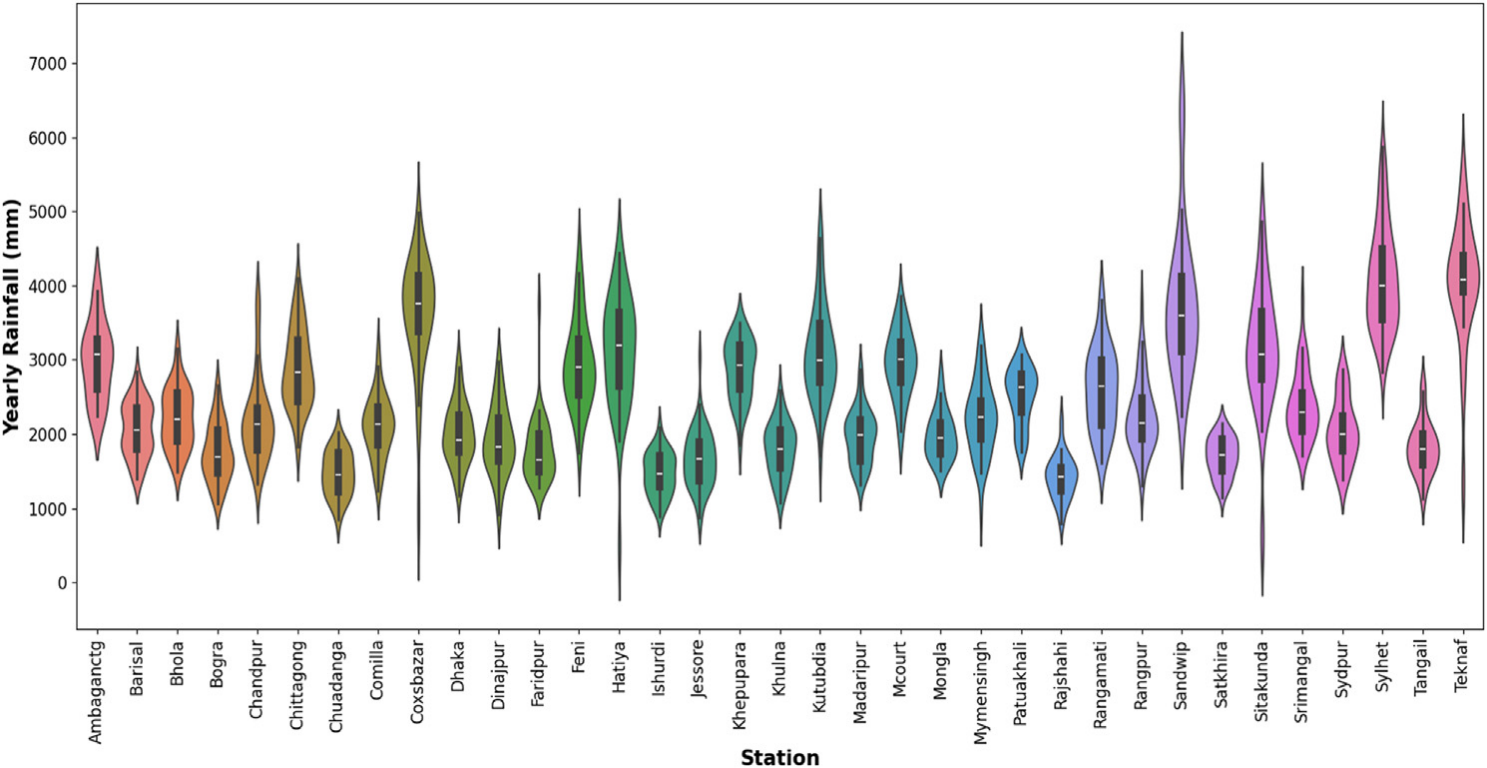
Comprehensive Violin Plot depicting annual rainfall (mm) distribution across 35 weather stations over the previous years.

Data analysis provides insight into the data and reveals the underlying structures and characteristics of a dataset. We are working with the data collected from 35 weather stations and each station has its own weather patterns. We have shown the weather trend (Dhaka weather station) in Fig. 7 and the distribution of rainfall in Fig. 8.

Fig. 7 illustrates the annual weather trend recorded in a single weather station in Dhaka over a decade. The data reveals a seasonal pattern with distinct and consistent changes in weather conditions observed over each year. For example, in Fig. 7(a), the humidity level shows the same recurring pattern for each year which implies the cyclical nature of the region's climate. The other three features show the same pattern throughout the decades.

The violin plot in Fig. 8 visualizes the annual rainfall distribution on a millimeter scale for 35 stations and compares rainfall patterns in different locations. Most of the locations have had rainfall of around 2000 to 4000 mm over the years. Very few stations (like Coxʼs Bazar) have a wide range of rainfall amounts with a significant spread, especially on the higher end, suggesting a high variability in rainfall. Among other stations, some of the stations (Sylhet, Teknaf, and Sitakunda) show a relatively wide range, whereas Teknaf and Sylhet displaying a higher median and wider IQR (Interquartile Range).

On the contrary, some of the stations (Feni, Hatiya, and Rangamati) have higher rainfall distributions, while other stations (Hatiya and Rangamati) show a significant upper whisker length, indicating the presence of extreme values or outliers in their rainfall data. Additionally, stations like Khulna, Ishurdi, Jessore, and Rajshahi have relatively narrow violins, indicating a more consistent rainfall amount with less variability. Ambaganctg and Barisal - these stations have moderate variability and median values.

### 4.3 Experimental results

The validity and usability of a dataset depend on the performance of a model built on it. So, we have created a weather forecast model using a Bi-LSTM model that can forecast the weather (Rainfall, Temperature, Humidity, and Sunshine). We used 3 Bi-LSTM layers with a time-distributed layer for the predictive model having a window size of 365. The performance metrics of the model on the dataset are mentioned in Table 2.

**Table 2:** Sample results of performance metrics of Bi-LSTM forecast model on the weather dataset.

Station	MAE	MSE	MSLE	R^2^	SMAP
Faridpur	0.0435	0.0084	0.0123	0.981	0.0235
Chandpur	0.0125	0.0017	0.0114	0.972	0.0255
Feni	0.0325	0.0041	0.01	0.983	0.0741
Tangail	0.073	0.0022	0.013	0.985	0.0424

It seems the forecast model created on our dataset shows outstanding performance and signifies that our data quality is good enough for data analysis and real-world weather forecast models.

## Limitations

Though we have collected and curated the high-quality weather dataset, there are also some limitations of our datasets. In the future, we will try to resolve the issues. Some of the limitations are as follows.

• We obtained daily data from 35 weather stations in Bangladesh, collected at 24-h intervals. However, if the data had been collected at 1-h intervals, it would have been more effective for developing robust forecast models.
• Weather stations were established at different periods, so the start and end years of data collection differ between stations.
• The data is only collected from Bangladesh, it may not generalize well to other regions.
• We have incorporated four features of the weather data. Some other features like precipitation, UV index, etc. should also be added.

## Ethics Statement

The authors have read and followed the ethical requirements for publication in Data in Brief and confirmed that the current work does not involve human subjects, animal experiments, or any data collected from social media platforms.

## CRediT Author Statement

**Md Zubair:** Investigation, Methodology, Data curation, Data pre-processing, Supervision, Writing – original draft, Visualization. **Md. Nafiz Ishtiaque Mahee:** Methodology, Data curation, Data pre-processing, Visualization. **Khondaker Masfiq Reza:** Data Curation, Data pre-processing, Writing – original draft. **Md. Shahidul Salim:** Conceptualization, Validation, Writing – review & editing. **Nasim Ahmed:** Conceptualization, Writing – review & editing.

## Data Availability

Mendeley DataHigh Volume Real-World Weather Data (Original data).
